# Military sexual trauma and mental health counseling: Effects on resilience over time among recent‐era U.S. veterans

**DOI:** 10.1002/jts.23137

**Published:** 2025-03-06

**Authors:** Mary M. Mitchel, Ryan P. Chesnut, Keith R. Aronson, Daniel F. Perkins

**Affiliations:** ^1^ Clearinghouse for Military Family Readiness Pennsylvania State University University Park Pennsylvania USA; ^2^ Social Science Research Institute University Park Pennsylvania USA; ^3^ Department of Biobehavioral Health University Park Pennsylvania USA; ^4^ Department of Agricultural Economics Sociology, and Education University Park Pennsylvania USA

## Abstract

Military sexual trauma (MST) is prevalent and causes numerous deleterious effects on survivors. This study investigated the association between mental health counseling (MHC) and resilience among a large cohort of U.S. veterans who served in support of military operations in Iraq and Afghanistan following the September 11, 2001, terrorist attacks. Data were collected over 6.5 years (Wave 1: *n* = 9,566, Wave 8: *n* = 2,970). Female veterans who experienced sexual harassment, β = −.12, and both sexual harassment and unwanted sexual contact, β = −.21, had lower baseline resilience scores. For male veterans, sexual harassment, β = −.08; unwanted sexual contact, β = −.09; and both sexual harassment and unwanted sexual contact, β = −.12, were related to lower baseline resilience scores. For both female, β = −.46, and male veterans, β = −.57, MHC was negatively associated with baseline resilience; however, MHC was positively associated with resilience scores over time for female, β = .17, and male veterans, β = .29. In the full mediation models tested, MHC mediated the path between all types of MST and resilience among male and female veterans. The findings suggest that engaging in MHC during the transition from active duty to civilian life may effectively increase resilience for veteran survivors of MST.

Military sexual trauma (MST) is defined in U.S. Code 1720D (Counseling and Treatment for Sexual Trauma, 2021) as:

Physical assault of a sexual nature, battery of a sexual nature, or sexual harassment (unsolicited verbal or physical contact of a sexual nature which is threatening in character) which occurred while the former member of the Armed Forces was serving on duty, regardless of duty status or line of duty determination.

MST includes being pressured into sexual activities, sexual contact, sexual activities without one's consent (e.g., being forced to have sex, unwanted sexual touching, any sexual activities that are threatening), and sexual harassment. MST is associated with psychological trauma and may result in physical injury (Wilson, 2018). MST can happen while a service member is on‐ or off‐base.

Prevalence estimates of MST vary widely across studies and have notable limitations (Wilson, 2018). In the U.S. Department of Defense's (DoD) annual survey, the prevalence of unwanted sexual contact among active duty service members was nearly 6.8% for female service members and 1.3% for male service members. In comparison, the survey found that the prevalence of sexual harassment was 24.7% and 5.8% for female and male service members, respectively. These prevalence rates tend to be higher for veterans. The U.S. Department of Veterans Affairs (VA) estimates that approximately one third of female and 2% of male veterans report being a survivor of MST (VA, 2021). A meta‐analysis found that nearly 16% of military personnel and veterans reported MST; the prevalence was 3.9% for men and 38.4% for women when sexual assault and harassment were included (Wilson, 2018).

As is well‐documented in the research literature, MST has deleterious effects on veterans’ health and well‐being (see for example, Yancey et al., 2024). In one study, individuals who screened positive for having experienced MST were 3 times more likely to have a mental health condition than those who screened negative (Kimerling et al., 2010). In addition, individuals who report experiencing MST have been found to be significantly more likely to develop posttraumatic stress disorder (PTSD; Kimerling et al., 2010; Sumner et al., 2021), depressive disorders (Kimerling et al., 2010), non‐PTSD–related anxiety disorders (Gross et al., 2019), and substance use disorders (Kimerling et al., 2010), and to attempt and die by suicide (Blais & Monteith, 2019). MST may also affect physical health. Sumner et al. (2021) examined national VA electronic medical records and found that individuals who experienced MST had increased odds of being diagnosed with virtually every physical health problem examined in the study.

Resilience is defined as a cognitive or emotional coping process an individual uses in response to a crisis or significant challenge that results in the individual recovering or rebounding to their precrisis functional level (Seery, 2011). The “stress inoculation theory” posits that exposure to stress can help individuals develop psychological resilience. In the face of stress, individuals may undergo psychological changes to accommodate and manage stressors more adeptly. Military service, while challenging and stressful, can build service members’ competence, coping skills, and courage. Indeed, individuals within the military often demonstrate resilience even in the face of life‐threatening and traumatic situations (Isaacs et al., 2017). Trauma and stress exposure are associated with personal resilience (Harms, 2015), and these exposures may contribute to higher levels of resilience within an individual (Isaacs et al., 2017).

Competing trauma theories suggest that exposures can leave individuals psychologically depleted and at heightened risk for poor coping and functioning. The “conservation of resources” theory posits that trauma often results in the loss (i.e., depletion) of one's resources (Hobfoll, 1989). Resources include social support, coping capacity, and a sense of safety. Psychologically, trauma is associated with negative changes in the survivor's cognitive schema and cognitive processing, which can lead to the further depletion of coping capacity (Janoff‐Bulman, 2010). Exposure to trauma, particularly repeated exposure, is associated with posttraumatic decline, which, in turn, can result in the complete or near complete depletion of a trauma survivor's psychological infrastructure and lead to a downward spiral in functioning.

For individuals who have experienced sexual trauma, professional assistance, such as mental health counseling (MHC), may lead to a stronger sense of resilience. MHC can help survivors understand what happened to them, adapt to their new normal, and move forward in life. Various MHC approaches have been successfully used among trauma survivors (Pond et al., 2023). These approaches include the use of cognitive behavior, acceptance and commitment, emotional disclosure, skills training, and/or feminist therapies. Irrespective of the type of MHC approach used, evidence suggests factors common to MHC and psychotherapy (e.g., positive regard, reflective listening) are the elements of MHC that are most salient in helping individuals obtain positive outcomes (Rubenstein et al., 2024). The DoD and VA offer in‐person and virtual MHC and therapy, sexual assault coordinators, victim advocates, and confidential help lines. There is evidence that a positive MST screen increases the likelihood of mental health treatment (Kimerling et al., 2007).

The current study examined the prevalence and correlates of MST in a large cohort of veterans who served in support of operations in Afghanistan and Iraq following the September 11, 2001, terrorist attacks (9/11) at baseline and over the first 6.5 years of their military‐to‐civilian transition. Based on the conservation of resources theory (Hobfoll, 1989), we hypothesized that MST would be negatively associated with resilience at baseline and over time. We also hypothesized that MHC would mediate the association between MST and resilience such that (a) veterans who were exposed to MST would be more likely to use MHC and (b) MHC would be associated with steeper increases in resilience when compared with veterans who did not utilize MHC.

## METHOD

### Participants and procedure

Data for the present study come from the Veterans Metric Initiative (TVMI) and the Veterans Engaging in Transitions Study (VETS). TVMI was a large‐scale, longitudinal, survey‐based study of post‐9/11 veterans’ separation from service and reintegration into civilian life. Details on the study design are published elsewhere (Vogt et al., 2018). In brief, this study used the VA/DoD Identity Repository (VADIR) to determine the population of all veterans who had separated from the military within the prior 3 months between August and November 2016 (*N* = 48,965). Approximately 19.5% (*n* = 9,566) of these individuals completed the baseline survey (see Table 1 for demographic characteristics). Surveys were readministered every 6 months over 2.5 years for a total of six waves of data collection. Retention rates for Waves 2–6 are as follows: 75.3% for Wave 2 (*n* = 7,200), 75.3% for Wave 3 (*n* = 7,201), 67.7% for Wave 4 (*n* = 6,480), 61.1% for Wave 5 (*n* = 5,844), and 55.0% for Wave 6 (*n* = 5,258).

VETS extended TVMI by collecting two additional waves of data from a subsample of veterans who agreed to be contacted about future research. Over one third of the original TVMI sample (*n* = 3,516, 36.8%) indicated an interest in continued research, and 3,180 individuals completed the Wave 7 survey, which was administered approximately 4 years postseparation. The Wave 8 survey was administered approximately 6.5 years postseparation, and 2,970 veterans (31.0%) completed this survey. In general, the VETS sample mirrored the baseline TVMI sample with regard to key demographic and military characteristics, such as biological sex, race, and pay grade. Ethical approval for TVMI was obtained from ICF International, Inc., and ethical approval for VETS was obtained from the Pennsylvania State University Internal Review Board (IRB). Informed consent was obtained prior to beginning the Wave 1 and Wave 7 surveys. Approval for VETS Wave 8 was obtained from the Pennsylvania State University IRB.

### Measures

#### Demographic characteristics

Demographic variables included service branch, pay grade, discharge status, military occupation, race/ethnicity, and marital status. The survey asked about biological sex, hence the study reports the results for male and female veterans.

#### Resilience

Resilience was measured using the six‐item Brief Resilience Scale (BRS; Smith et al., 2008), which was administered at Waves 1, 5, and 8 (sample items: “You tend to bounce back quickly after hard times,” “It is hard for you to snap back when something bad happens”). Response options range from 1 (*strongly disagree*) to 5 (*strongly agree*). Three negatively worded items (Items 2, 4, and 6) are reverse‐scored. Items are then summed, with total scores ranging from 6 to 30 and higher scores indicating higher levels of resilience. In the current study, the averages of the resilience scale scores were used. Cronbach's alphas were .88 at Wave 1, .78 at Wave 5, and .81 at Wave 8.

#### MST

Military sexual trauma (MST) was measured at Wave 1 using the two‐item MST Screener (Kimerling et al., 2007), which retrospectively assesses respondents’ experiences of military service–related sexual harassment (i.e., “When you were in the military, did you ever receive unwanted, threatening, or repeated sexual attention?”) and assault (i.e., “When you were in the military, did you have sexual contact against your will or when you were unable to say no?”). Prior studies have provided evidence supporting the screener's validity in male and female veteran samples (Kimerling et al., 2007). Respondent answers were coded as (a) no MST, (b) sexual harassment alone, (c) sexual assault alone, or (d) both sexual harassment and assault. Internal consistency was high, with a Chronbach's alpha of .97.

#### MHC

MHC was measured at Wave 1. Participants were presented with a single item developed for TVMI that inquired about the frequency with which they had used MHC services over the prior 3 months. Answers were recorded on a 6‐point Likert‐type scale with response options of 0 (*never*), 1 (*less than once a month*), 2 (*once or twice a month*), 3 (*3 or 4 times a month*), 4 (*2 to 3 times a week*), and 5 (*4 or more times a week*). The MHC variable was positively skewed (skewness > 3.0); thus, we dichotomized responses into 0 (never) versus 1 (once or more).

#### Covariates

There were two covariates in this study. Combat exposure (CE) was measured at Wave 1 using nine items from the Deployment Risk and Resilience Inventory–2 (DRRI‐2; Vogt et al., 2013) Combat Exposure and Aftermath of Battle scales. Respondents indicated the frequency with which they encountered combat‐related events (e.g., “During combat, how often did you fire your weapon at enemy combatants?”) and witnessed the aftermath of combat (e.g., “During combat, how often did you see civilians after they had been severely wounded or disfigured?”). Responses were recorded on a Likert‐type scale with response options ranging from 0 (*never*) to 3 (*many times*). The DDRI‐2 has demonstrated reliability and validity in veteran samples (Bovin et al., 2022; Vogt et al., 2013), and the results of a recent study of Iraq and Afghanistan veterans confirmed the measure's reliability and validity (Bovin et al., 2023). Because The sum score was positively skewed (skewness > 1.0); thus, we dichotomized responses into 0 (never) versus 1 (one or more events).

#### Adverse childhood experiences

Adverse childhood experiences (ACEs) were measured at Wave 5 with a seven‐item questionnaire developed for a health‐related study of U.S. Marine recruits (LeardMann et al., 2010). The measure asks participants to indicate (i.e., “yes” or “no”) if they experienced physical neglect, physical abuse, emotional neglect, emotional abuse, domestic violence, sexual abuse, or a family history of mental health challenges or substance abuse before the age of 17 years. Previous research using veteran samples has produced evidence substantiating the validity of the seven‐item scale (Doucette et al., 2023). Items were summed to create a count of ACEs and then trichotomized to reflect zero ACEs, one or two (1–2) ACEs, and three or more (≥ 3) ACEs.

### Data analysis

After the frequencies and means were generated for all study variables for male and female veterans, an unconditional multiple group (male vs. female) latent growth curve model (Curran, 2000) was fit using the averaged resilience scale scores at baseline (i.e., within 3 months of separation) and 2 years and 6.5 years following separation from the military. Because there were missing data over time that were handled using the auxiliary function in *Mplus*, the maximum likelihood robust estimator that employs full information maximum likelihood was used for the unconditional and conditional models, and the weighted least squares with mean and variance–adjusted estimator was used to fit the mediation models. Model fit was assessed using conventional guidelines (Hu & Bentler, 1999), including comparative fit index (CFI) and Tucker–Lewis index (TLI) values of .95 or higher, a root mean square error of approximation (RMSEA) value of .06 or less, and a standardized root mean square residual (SRMR) value of .08 or less.

The study met the recommended criteria for fitting a latent growth model (e.g., a sample size well above the recommended criterion of 100 participants; see Curran, 2000). There were three repeated measures, meeting the minimum for linear growth curve modeling, and maximum likelihood estimation was used for the unconditional and main effects models given that the mean resilience scores at each of the three waves were approximately normally distributed with only a slight skew value ranging from 0.30 to −0.36 over this period.

We fit a mediation model that used the multiple group latent growth model for female (*n* = 1,741) and male veterans (*n* = 7,811), with limited missing observations (*n* = 14) due to missing all study variables (*n* = 2) or missing the CE variable (*n* = 12). The direct and indirect effects paths from MST variables to MHC (i.e., *a* path) and from the latter to the slope latent variable (i.e., *b* path) along with the indirect effect (i.e., a*b) were tested using bootstrapping with 10,000 draws for assessing the 95% confidence intervals (CIs) associated with the paths.

Missing data due to sample attrition were handled using the auxiliary variable function in *Mplus* (Version 8; Muthen & Muthen, 2017). Variables that were correlated with missing values at baseline (Vogt et al., 2018) were included in the auxiliary variable function to make the dataset better fit the missing at random (MAR) assumption. Achieving MAR increases power and reduces bias. The auxiliary variables included age, race, education, pay grade, marital status, National Guard or Reserve status, medical insurance, and home address. Education and pay grade are proxy measures for socioeconomic status (SES).

A sample weight, calculated using Wave 1 variables, was applied to adjust the results based on the sample respondents’ proportions versus the general population characteristics of post‐9/11 veterans from 2016. The sample weights were calculated based on the gender, pay grade, and service branch proportions of veterans in the larger population of post‐9/11 veterans from which the TVMI sample was drawn (Vogt et al., 2018).

## RESULTS

### MST frequency

Results revealed that 38.0% (*n* = 677) of female veterans self‐reported experiencing unwanted sexual attention, and 16.5% (*n* = 288) self‐reported experiencing unwanted sexual contact. Approximately 3.0% of the male veterans (*n* = 237) self‐reported experiencing unwanted sexual attention and 1.2% (*n* = 90) reported unwanted sexual contact.

### Resilience

Mean resilience scores over the three waves ranged from 3.4 to 3.6 for female veterans (i.e., a medium level of resilience), with standard deviations of 0.8 to 0.9. Mean resilience scores for men fell in the medium‐high range, with scores ranging from 3.7 to 3.8 with a standard deviation of 0.8 for all three time points (see Table 1).

**TABLE 1:** Comparison of sample characteristics between female and male veterans

	Female veterans		Male veterans		
	(*n* = 1,743)		(*n* = 7,823)		
Variable	*n*	%	*n*	%	*p* ^a^
Race/ethnicity					< .001
White	1,061	60.9	5,765	73.7	
African American/Black	329	18.9	737	9.4	
Other	351	20.2	1,316	16.8	
Paygrade					< .001
E1–E4	541	31.0	2,163	27.6	
E5–E6	532	30.5	2,339	29.9	
E7–E9	242	13.9	1,466	18.7	
W1–W5 and O1–O3	205	11.8	745	9.5	
O4–O10	223	12.8	1,109	14.2	
Adverse childhood experiences^b^					< .001
0	443	41.2	2,902	60.4	
1–2	288	26.8	1,037	21.6	
≥ 3	345	32.1	862	18.0	
Combat exposure					< .001
0	1,148	65.9	3,303	42.3	
≥ 1	593	34.1	4,509	57.7	
Use of MHC					0.001
Never	1,468	85.1	6,734	87.6	
Once or more	258	14.9	949	12.4	
MST				,	< .001
No MST	1,041	59.8	7,555	96.7	
Sexual harassment	413	23.7	169	2.2	
Sexual Assault	24	1.4	22	0.3	
Both sexual harassment and sexual assault	264	15.2	68	0.9	

	*M*	*SD*	*M*	*SD*	*p* ^c^
Age (years)	33.1	9.3	34.8	9.6	< .001
Resilience score^d^					
Wave 1	3.6	0.8	3.8	0.8	< .001
Wave 5	3.5	0.9	3.8	0.8	< .001
Wave 8	3.4	0.8	3.7	0.8	< .001

*Note*: MHC = mental health counseling; MST = military sexual trauma.

^a^ Calculated based on chi‐square tests.

^b^ Measured at Wave 5 (female: *n* = 1,076, male: *n* = 4,801).

^c^ Calculated based on *t* tests.

^d^ Range: 1–5.

### Unconditional multiple group latent growth curve model

In latent growth curve analysis, unconditional models are constructed to examine the covariances among the observed variables compared to the model‐implied covariances and include the entire sample without any predictors. The unconditional model achieved excellent model fit, CFI = 1.00, TLI = .99, RMSEA = .02, 90% CI [0.01, 0.04], SRMR = 0.01, χ^2^(2, N *=* 9,563) = 7.33, *p* = .027. Next, we examined the variances of the latent growth factors (i.e., resilience at baseline and over time). The results revealed significant variance for both latent growth factors for male and female veterans, *p* < .001. For women, the mean resilience score at baseline was 3.56, *p* < .001, and resilience declined slightly over time (i.e., −0.07), *p* < .001. For men, the mean resilience score at baseline was 3.77, *p* < .001, and resilience declined slightly over time (i.e., −0.04), *p* < .001. Taken together, resilience scores slightly declined over time in the full sample.

### Associations among MST, ACEs, CE, and resilience at baseline and over time

A multiple group (i.e., all men vs. all women in the sample) conditional model with predictor variables of MST, ACEs, and CE achieved good model fit, CFI = .96; TLI = .95; RMSEA = .03, 90% CI [0.03, 0.03]; SRMR = 0.02, χ^2^(30, *N* = 9,551) = 151.95, *p* < .001. This model revealed that female veterans who self‐reported experiencing sexual harassment, *B* = −0.19, *SE* = 0.05, β = −.12, *p* < .001, or both sexual harassment and unwanted sexual contact, *B* = −0.40, *SE* = 0.06, β = −.21, *p* < .001, had lower resilience scores at baseline compared to female veterans who self‐reported no MST (Table 2). Female veterans who self‐reported experiencing three or more ACEs had lower baseline resilience scores, *B* = −0.28, *SE* = 0.06, β = −.20, *p* < .001, than those who self‐reported no ACEs. However, no predictors were associated with the change in resilience scores over time (Table 2).

**TABLE 2:** Associations among military sexual trauma, combat exposure, adverse childhood experiences

	Female veterans				Male veterans			
	(*n* = 1,741)				(*n* = 7,810)			
Variable	*B*	*SE*	β	*p*	*B*	*SE*	β	*p*
Resilience intercept regressed on:								
SH	−0.19	0.05	−.12	< .001	−0.34	0.07	−0.08	< .001
SA	−0.12	0.14	−.02	.375	−1.06	0.17	−0.09	< .001
Both SH and SA	−0.40	0.06	−.21	< .001	−0.77	0.14	−0.12	< .001
Combat exposure (yes)	−0.06	0.04	−.04	.146	−0.04	0.02	−0.03	.033
ACEs (0)	Ref.	Ref.						
ACEs (1–2)	−0.11	0.06	−.07	.082	−0.17	0.03	−0.11	< .001
ACEs (≥ 3)	−0.28	0.06	−.20	< .001	−0.30	0.03	−0.17	< .001
Resilience slope regressed on:								
SH	−0.02	0.03	−.05	.429	0.06	0.03	0.05	.044
SA	0.14	0.08	.08	.077	0.55	0.21	0.16	.008
Both SH and SA	0.05	0.04	.09	.166	1.16	0.58	0.11	.048
Combat exposure (yes)	0.03	0.02	.08	.151	0.04	0.01	0.10	< .001
ACEs (0)	Ref.				Ref.			
ACEs (1–2)	−0.02	0.03	−.05	.514	−0.001	0.01	−0.003	.924
ACEs (≥ 3)	−0.01	0.03	−.02	.805	0.01	0.02	0.02	.465

*Note*: Fit statistics: Comparative fit index = .96; Tucker–Lewis Index = .95; root mean square error of approximatio*n* = .03, 90% confidence interval [0.03, 0.03]; standardized root mean square residual = .02; χ^2^(30) = 151.95, *p* < .001. SH = sexual harassment; SA = sexual assault; Ref. = reference category; ACEs = adverse childhood experiences.

For male veterans, self‐reported sexual harassment, *B* = −0.34, *SE* = 0.07, β = −.08, *p* < .001; unwanted sexual contact, *B* = −1.06, *SE* = 0.17, β = −.09, *p* < .001; both sexual harassment and sexual assault, *B* = −0.77, *SE* = 0.14, β = −.12, *p* < .001; CE, *B* = −0.04, *SE* = 0.02, β = −.03, *p = *.033; one to two ACEs, *B* = −0.17, *SE* = 0.03, β = −.11, *p* < .001; or three or more ACEs, *B* = −0.30, *SE* = 0.03, β = −.17, *p* < .001, were related to lower baseline resilience scores (Table 2). Self‐reported sexual harassment, *B* = 0.06, *SE* = 0.03, β = .05, *p* = .044; unwanted sexual contact, *B* = 0.55, *SE* = 0.21, β = .16, *p* = .008; both sexual harassment and unwanted sexual contact, *B* = 1.16, *SE* = 0.58, β = .11, *p* = .048; and CE, *B* = 0.04, *SE* = 0.01, β = .10, *p* < .001, were positively associated with change in resilience scores over time.

Compared to female veterans who self‐reported experiencing sexual harassment, *B* = −0.02, *SE* = 0.03, β = −.05, *p* = .429, male veterans who self‐reported experiencing sexual harassment demonstrated a significant increase in resilience over time, *B* = 0.06, *SE* = 0.03, β = .05, *p = *.044, with a significant Wald test, *p* = .044. Resilience did not change over time among female veterans who self‐reported experiencing sexual harassment. Male veterans who self‐reported experiencing unwanted sexual contact, *B* = −1.06, *SE* = 0.17, β = −.09, *p* < .001, had significantly lower baseline resilience scores, with a significant Wald test, *p* < .001, compared to female veterans who self‐reported experiencing unwanted sexual contact, *B* = −0.12, *SE* = 0.14, β = −.02, *p* = .375. Male veterans who self‐reported experiencing both sexual harassment and unwanted sexual contact, *B* = −0.77, *SE* = 0.14, β = −.12, *p* < .001, had significantly lower baseline resilience scores compared to female veterans who self‐reported experiencing both sexual harassment and unwanted sexual contact, *B* = −0.40, *SE* = 0.06, β = −.21, *p* < .001, with a significant Wald test, *p* = .012.

### Mediation model with MST, MHC, and resilience

The mediation model assumed that MST occurred prior to engaging in MHC. Respondents were asked about MHC attendance within the past 3 months. Based on the sampling procedure, this time horizon would have occurred after separation from the military. In turn, the mediation model tested the association between MHC and resilience over time. The model achieved adequate fit, CFI = .94; TLI = .89; RMSEA = .03, 90% CI [0.03, 0.04]; χ^2^(38, *N* = 9,552) = 245.28, *p* < .001. As shown in Table 3, female, *B* = 0.34, *SE* = 0.09, β = .14, 95% CI [0.07, 0.21], and male veterans, *B* = 0.36, *SE* = 0.11, β = .05, 95% CI [0.02, 0.08], who self‐reported experiencing sexual harassment were significantly more likely to obtain MHC than peers who did not self‐report such experiences. Female, *B* = 0.74, *SE* = 0.10, β = .26, 95% CI [0.19, 0.32], and male veterans, *B* = 0.68, *SE* = 0.17, β = .06, 95% CI [0.03, 0.09], who self‐reported experiencing both sexual harassment and sexual assault were also significantly more likely to attend MHC, as were male veterans who self‐reported experiencing sexual assault, *B* = 0.76, *SE* = 0.31, β = .04, 95% CI [0.01, 0.07].

**TABLE 3:** Military sexual trauma, mental health counseling (MHC), and resilience, at baseline and over time

	Female veterans				Male veterans			
	(*n* = 1,741)				(*n* = 7,811)			
Variables	*B*	*SE*	β	*p*	*B*	*SE*	β	*p*
Resilience intercept regressed on:								
SH	−0.12	0.05	−.07	.018	−0.24	0.07	−.05	< .001
SA	−0.11	0.22	−.02	.611	−0.86	0.26	−.07	.001
Both SH and SA	−0.24	0.06	−.12	< .001	−0.51	0.11	−.07	< .001
Combat exposure yes	−0.07	0.04	−.05	.092	−0.07	0.02	−.05	< .001
ACEs 0	Ref.				Ref			
ACEs 1–2	−0.13	0.08	−.08	.092	−0.19	0.04	−.12	< .001
ACEs ≥ 3	−0.31	0.07	−.21	< .001	−0.33	0.04	−.19	< .001
MHC yes	−0.31	0.03	−.46	< .001	−0.37	0.01	−.57	< .001
Resilience slope regressed on:								
SH	−0.03	0.03	−.05	.365	0.04	0.03	.03	.221
SA	0.20	0.10	.12	.049	0.34	0.24	.10	.162
Both SH and SA	0.06	0.04	.11	.106	0.11	0.08	.06	.176
Combat exposure yes	0.03	0.02	.06	.258	0.04	0.01	.10	.001
ACEs 0	Ref.				Ref.			
ACEs 1–2	0.01	0.04	.02	.800	0.003	0.05	.01	.954
ACEs ≥ 3	0.02	0.03	.05	.526	0.02	0.03	.04	.492
MHC yes	0.03	0.02	.17	.048	0.05	0.01	.29	< .001
MHC regressed on:								
SH	0.34	0.09	.14	< .001	0.36	0.11	.05	.001
SA	0.16	0.74	.02	.827	0.76	0.31	.04	.014
Both SH & SA	0.74	0.10	.26	< .001	0.68	0.17	.06	< .001

*Note*: *N* = 9,552. SH = sexual harassment; SA = sexual assault; ACEs = adverse childhood experiences; Ref. = reference category.

For both female, *B* = −0.31, *SE* = 0.03, β = −.46, 95% CI [−0.54, −0.37], and male veterans, *B* = −0.37, *SE* = 0.01, β = −.57, 95% CI [−0.60, −0.53], MHC was negatively associated with baseline resilience. However, MHC was positively associated with resilience scores over time for female, *B* = 0.03, *SE* = 0.02, β = .17, 95% CI [−0.01, 0.35], and male veterans, *B* = 0.05, *SE* = 0.01, β = .29, 95% CI [0.19, 0.39].

### Mediation Effects in the association between MST and resilience

**FIGURE 1:**
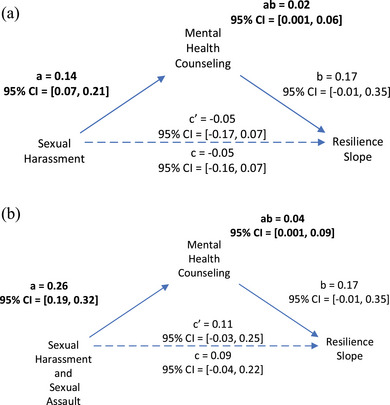
Mental health counseling (MHC) as a mediator of the association between military sexual trauma (MST) types and resilience over time among female veterans exposed to (A) sexual harassment and (B) both sexual harassment and sexual assault *Note*: Data were derived from 1,741 female veterans. Bolded path coefficients indicate significant direct or indirect effects (i.e., bootstrapped 95% confidence intervals [CI] do not include 0). Solid lines with arrows indicate significant paths, and dotted lines with arrows represent nonsignificant paths.

Figure 1A illustrates that a significant mediational effect occurred for MHC in explaining the association between self‐reported sexual harassment and resilience such that female veterans who self‐reported experiencing sexual harassment were more likely to engage in MHC, and, in turn, MHC was associated with increased resilience over time, standardized mediation effect (SME)_a*b_ = .02, 95% CI [.001, .06]. Figure 1B shows a significant indirect effect of MHC as a mediator in the association between self‐reported experiences of both sexual harassment and unwanted sexual contact and increasing resilience over time, SME_a*b_ = .04, 95% CI [.001, .09].

**FIGURE 2:**
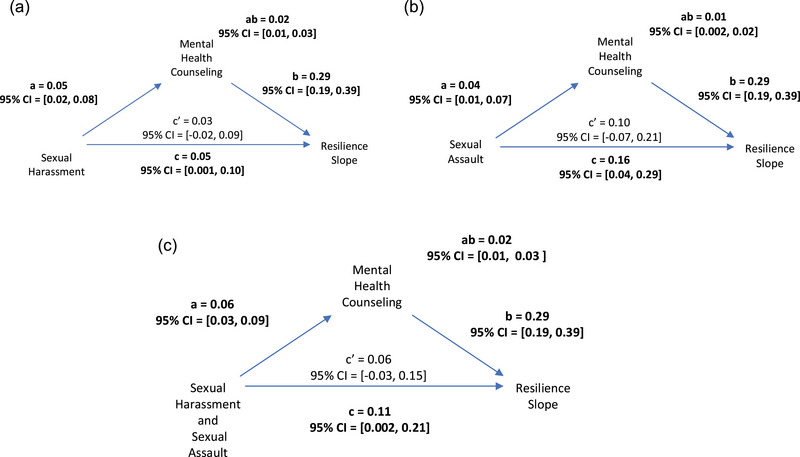
Mental health counseling (MHC) as a mediator of the association between military sexual trauma (MST) types and resilience over time among male veterans exposed to (A) sexual harassment, (B) sexual assault, and (C) both sexual harassment and sexual assault *Note*: Data were derived from 7,811 male veterans. Bolded path coefficients indicate significant direct or indirect effects (i.e., bootstrapped 95% confidence intervals [CI] do not include 0). Solid lines with arrows indicate significant paths, and dotted lines with arrows represent nonsignificant paths.

Significant mediational effects occurred for MHC explaining the association between all three MST predictors and resilience slopes for male veterans. As shown in Figure 2A, men who self‐reported experiencing sexual harassment were more likely to engage in MHC, which, in turn, was associated with a significant increase in resilience over time, SME_a*b_ = .02, 95% CI [.01, .03]. Figure 2B shows that men who self‐reported experiencing sexual assault were more likely to engage in MHC, and, in turn, MHC was associated with steeper increases in resilience over time, SME_a*b_ = .01, 95% CI [.001, .02]. Figure 2C shows that male veterans who self‐reported experiencing both sexual harassment and unwanted sexual contact were more likely to engage in MST, which, in turn, was associated with increased resilience scores over time, SME_a*b_ = .02, 95% CI [.01, .03].

## DISCUSSION

This study examined MST experiences in a large sample of post‐9/11 veterans over the first 6.5 years of transitioning from military to civilian life. Very little is known about how veteran transitioning unfolds over time and how veterans change during this period. The lack of understanding is particularly striking for female veterans, who are often not included in studies of military‐to‐civilian transitions.

Trauma exposure of a sexual nature (e.g., MST), particularly when perpetrated by trusted individuals (e.g., military comrades), can have long‐lasting effects on survivors (Kimerling et al., 2010). Survivors who are able to seek and receive counseling may have different healing trajectories compared to those who do not receive assistance (Harms, 2015). However, it is important to note that both admitting to and healing from trauma is often very difficult, even in cases where individuals access high‐quality support systems (O'Brien et al., 2015). Thus, it was imperative to examine whether survivors of MST were more likely to engage in MHC and if MHC was, in turn, associated with steeper increases in resilience over time.

In line with prior research (Kimerling et al., 2010; Wilson, 2018), we found that female veterans were substantially more likely than their male peers to self‐report MST exposure, with nearly 40% of women in the sample self‐reporting unwanted sexual attention and 17% reporting unwanted sexual contact. Approximately 3% and 1% of male veterans self‐reported being survivors of unwanted sexual attention and sexual contact, respectively. Some research has suggested that MST may be underreported due to perceived stigma, potential victim‐blaming, and fear of reprisal (Acosta et al., 2021).

The findings partially supported our hypothesis that veterans who self‐reported MST exposure would have lower resilience scores at baseline compared to peers who did not self‐report such experiences. Compared to female veterans who did not endorse MST exposure, those who self‐reported experiencing sexual harassment and a combination of sexual harassment and unwanted sexual contact reported lower levels of resilience at baseline. Female veterans who self‐reported experiencing unwanted sexual contact, CE, or one to two ACEs did not evidence lower resilience scores at baseline. There may have been insufficient power to find an MST effect, as the sample size for women who self‐reported unwanted sexual contact was small (*n* = 24).

A somewhat different profile emerged for male veterans. Compared to men who did not endorse MST exposure, those who self‐reported experiencing sexual harassment, unwanted sexual contact, and/or a combination of sexual harassment and unwanted sexual contact had lower levels of resilience at baseline. In addition, male veterans with CE and any self‐reported ACEs had lower levels of baseline resilience compared to those with no CE or ACEs. It is possible that cultural beliefs that sexual trauma cannot victimize men may obstruct their recovery, and this situation could lead to feelings of inadequacy, guilt, and shame (O'Brien et al., 2015); research suggests that these feelings may be particularly pronounced in the military.

For men, all forms of self‐reported MST were significantly and positively associated with steeper increases in resilience compared to men who did not self‐report such experiences. One explanation for this finding is that male veterans who self‐reported experiencing MST evidenced lower resilience scores at baseline and, thus, had more room to increase their scores. In contrast, men who did not self‐report any MST experiences may have had a ceiling effect. In addition, the challenges and stressors associated with military life and the competence, coping skills, and courage that military service can engender may lead to an ability to demonstrate resilience in male service members, as noted by stress inoculation theory (Meichenbaum & Fitzpatrick, 1993). For female veterans, less steep increases in resilience over time may reflect their increased risk of being exposed to trauma revictimization. Female military personnel are more likely to have experienced childhood trauma than their male peers, which increases the likelihood of comorbid mental health concerns among women in the military (Doucette et al., 2023). The cumulative impact of traumatic experiences among women may make it more difficult for them to build resilience (Bourke, 2022).

To investigate these findings further, we fit mediation models to determine whether obtaining MHC could explain the association between MST exposure and reporting faster growth in resilience over time. Compared to veterans who did not self‐report MST exposure, those with self‐reported MST exposure were more likely to engage in MHC, and the results indicate that participating in MHC, regardless of dosage, was conducive to building resilience more quickly compared to not engaging in MHC. Over time, MHC was positively associated with resilience for female and male veterans, indicating that resilience growth was faster among veterans who experienced MST and obtained MHC compared with their peers who did not experience MST. Furthermore, numerous MHC strategies have been shown to bolster one's ability to deal with trauma (Joyce et al., 2018). For example, cognitive behavioral therapy has been shown to effectively increase resilience among veterans (Baek et al., 2024).

There were several study limitations to note. First, no information was collected regarding the types of MHC veterans received or whether it was evidence‐informed, nor were we able to assess the quality with which MHC was delivered. Therefore, this study could not describe which approaches were successful and which were not. In addition, the survey only asked about attending MHC at Wave 1 (i.e., within 3 of separation from the military). Thus, some veterans may have received more MHC during their transition. Nonetheless, even without a particular evidence‐based or technique‐based approach to MHC, common factors are typically the strongest predictors of patient outcomes (Messer & Wampold, 2002). Common factors in MHC include the therapeutic alliance, decreased social isolation, the provision of information, and the instillation of hope (Tschacher et al., 2014). Second, the sample size of female veterans who experienced only unwanted sexual contact was small. This likely limited our ability to detect MHC effects for this group. On the other hand, the effect sizes found in this study were small. As a result, the clinical utility of the findings is limited. Future studies would benefit by recruiting larger samples of veteran survivors of unwanted sexual contact for a more robust evaluation of the associations between MHC and the outcomes examined here. Third, resilience was only measured at three assessment points, which limited our ability to detect any nonlinear effects of MHC on resilience. The findings of this study should not be generalized to all veterans, although TVMI is largely representative of post‐9/11 veterans who left service in 2016. Fourth, the use of the two‐item measure of MST was not ideal, as more comprehensive measures of MST exist, and these measures may provide more precise estimates of MST. Indeed, Some research has suggested that the two‐item screener used in the VA likely misses cases (Wilson, 2018). In the present study, there was a difficult balance between using a more comprehensive measure of MST and reducing participant burden. Finally, numerous unmeasured variables may account for changes in resilience over time, including access to health care, community connections, employment, and financial stability.

This study attempted to shed light on the impact of MHC as a mediating variable that could explain the association between MST exposure that occurs during active duty and building resilience over the first 6.5 years of transitioning to civilian life. Unique to this study was the examination of the association between MHC and resiliency over time in a large sample of post‐9/11 veterans as they transitioned from military to civilian life. Military‐to‐civilian transitions are challenging for a significant minority of veterans and, hence, understanding resiliency during this period is important, particularly among individuals who may be vulnerable to more difficult transitions, such as those who have experienced MST or other traumatic events.

The study found support for the conservation of resources theory (Hobfoll, 1989) of trauma and resilience, which suggests that trauma exposure can be highly disruptive to one's sense of self and trust in the world. Importantly, the study also revealed that MHC was associated with increases in resilience among post‐9/11 veterans. As veterans transition to civilian life, they should be encouraged to seek MHC with VA or non‐VA clinicians; this is particularly important for veterans who may have an increased risk of experiencing problematic transitions (e.g., those who have experienced trauma exposure or combat, veterans with a high disability rating). Referrals to veteran‐serving organizations should also be provided to transitioning veterans, as these organizations offer a range of support services to help ease veteran reentry into civilian life. Finally, although the DoD has taken steps to prevent MST, few of these programs have been sufficiently evaluated to establish whether they are effective.

## AUTHOR NOTE

This work was supported by the Henry M. Jackson Foundation for the Advancement of Military Medicine (Grant #2021‐48709‐35659). The Veterans Metrics Initiative (TVMI) research was managed by the Henry M. Jackson Foundation for the Advancement of Military Medicine, Inc. (HJF), and it was collaboratively sponsored by the Bob Woodruff Foundation, Health Net Federal Services, HJF, Lockheed Martin Corporation, Marge and Philip Odeen, May and Stanley Smith Charitable Trust, National Endowment for the Humanities, Northrop Grumman, Prudential, Robert R. McCormick Foundation, Rumsfeld Foundation, Schultz Family Foundation, The Heinz Endowments, U.S. Department of Veterans Affairs Health Services Research and Development Service, Walmart Foundation, and Wounded Warrior Project, Inc. Support for the Veterans Engaging in Transition Studies (VETS) Wave 7 survey was provided by The Pew Charitable Trusts (Grant 34406). This work leverages funds from the USDA National Institute of Food and Agriculture and Hatch Appropriations (2021‐48709‐35659). Support for the Veterans Engaging in Transition Studies (VETS) Wave 8 survey was provided by was provided by the Wounded Warrior Project (265991), The Heinz Endowments (G0197), and May & Stanley Smith Charitable Trust and The Arthur M. Blank Family Foundation (76853129).

The views expressed are those of the author(s) and do not necessarily reflect the views of the Henry M. Jackson Foundation for the Advancement of Military Medicine, Pew Charitable Trusts, Wounded Warrior Project, Heinz Endowments, May & Stanley Smith Charitable Trust, or Arthur M. Blank Family Foundation.

## OPEN PRACTICES STATEMENT

The data that support the findings of Waves 1–6 of this study (The Veterans Metric Initiative [TVMI]) are openly available in the Inter‐University Consortium for Political and Social Research (ICPSR) at https://www.icpsr.umich.edu/web/ICPSR/studies/38051/publications, reference number (ICPSR 38051). The data that support the findings of Waves 7–8 (Veterans Engaging in Transition Studies [VETS]), an extension of the TVMI study that collected two additional surveys, are not yet publicly available but will be available at ICPSR within the next 18 months.
